# Gastrointestinal symptoms as the first manifestation of antiphospholipid syndrome

**DOI:** 10.1186/s12876-021-01736-2

**Published:** 2021-04-01

**Authors:** Xiaojuan Zou, Zhongqi Fan, Ling Zhao, Weiling Xu, Jin Zhang, Zhenyu Jiang

**Affiliations:** 1 Department of Rheumatology, The First Hospital of Jilin University, Changchun, 130012 Jilin China; 2 Department of Hepatobiliary Pancreatic Surgery, The First Hospital of Jilin University, Changchun, 130012 China; 3 Department of Radiology, The First Hospital of Jilin University, Changchun, 130012 China; 4 Department of Cardiovascular Center, The First Hospital of Jilin University, Changchun, 130012 Jilin China

**Keywords:** Antiphospholipid syndrome, Antiphospholipid antibodies, Intestinal necrosis, Intestinal obstruction, Mesenteric thrombosis

## Abstract

**Background:**

Antiphospholipid syndrome (APS) is an acquired pre-thrombotic autoimmune condition, which produces autoantibodies called antiphospholipid antibodies (APL) against phospholipid-binding plasma proteins. The diagnosis of APS requires at least one of Sapporo standard clinical manifestations and one laboratory criteria (persistently medium/high titer anticardiolipin antibodies, and/or medium/high titer anti-β2-glycoprotein I antibodies, and/or a positive lupus anticoagulant test). Gastrointestinal lesions are rarely reported in APS patients. APS cases with recurrent abdominal pain as the first clinical manifestation are even rarer.

**Case presentation:**

This report describes an APS case with recurrent abdominal pain as the first clinical manifestation of antiphospholipid syndrome. The patient has a history of two miscarriages. Computed tomography of the abdomen confirmed mesenteric thrombosis and intestinal obstruction while laboratory tests for serum antiphospholipid and anti-β2-glycoprotein I antibodies were positive. This led to the diagnosis of APS.

**Conclusions:**

This paper provides useful information on gastrointestinal manifestations and APS, also including a brief literature review about possible gastrointestinal symptoms of APS.

## Background

Antiphospholipid syndrome (APS) is an autoimmune disease characterized by persistent positive antiphospholipid antibodies (APL) in the bloodstream that leaves the patient in a potentially hypercoagulable state. This causes thrombus formation at all segments of the vascular bed [1]. Based on whether the APS is secondary to other autoimmune diseases, it is divided into primary and secondary: APS without other autoimmune diseases are called primary APS (PAPS). APS with systemic lupus erythematosus or other autoimmune diseases is called secondary APS (SAPS). Common clinically significant laboratory tests for APL include lupus anticoagulants (LA), anti-β2-glycoprotein I antibodies (anti-β2GPI) and anticardiolipin antibodies (ACL). For the diagnosis of APS, APL requires at least two positive tests at least 12 weeks apart to be meaningful, and at least one clinical presentation is required to meet updated Sapporo standards (Vascular thrombosis and/or Pregnancy morbidity) [2, 3].

APS-related manifestations of gastrointestinal tract have been reported successively, in esophagus, stomach, intestines, etc. [4, 5]. However, abdominal lesions are not prevalent in APS patients. Gastrointestinal manifestations are rarely reported in APS patients [6], APS cases with recurrent abdominal pain are more rarer.

Here, we report a case of APS with recurrent abdominal pain as the first symptom, who was admitted to a local hospital for recurrent abdominal pain in gastroenterology and other departments. The diagnosis was unclear. The aim of this case report is that other departments can strengthen their understanding of APS. These departments will be able to achieve early diagnosis and early treatment and be able to adopt an appropriate treatment plan according to relevant literature and guidelines. This will achieve good efficacy and reduce missed diagnosis.

## Case presentation

**Fig. 1:**
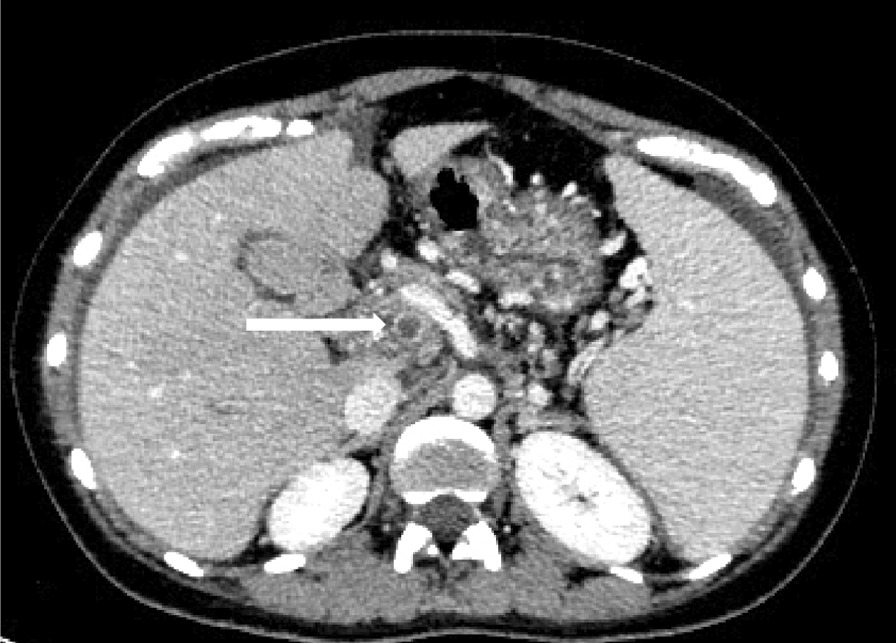
Multiple thrombosis of portal vein, splenic vein, superior mesenteric vein and some branches

A 32-year-old woman was admitted to the gastroenterology department for upper abdominal pain. Gastroenteritis was continually managed using omeprazole. The treatment relieved her symptoms just a little. The symptoms of upper abdominal pain appeared intermittently for about 8 months. Repeated visits to the gastroenterology department and other departments in the local hospital were made. Omeprazole treatment for gastroenteritis was continued. However, the symptoms were still not improving. Finally, the patient went to the local gastrointestinal surgery department. The abdomen computed tomography examination was performed, which revealed that thrombosis had occurred in the portal vein, superior mesenteric vein and splenic vein (Fig. 1). The thrombus did not improve even after repeated visits in gastrointestinal surgery and vascular surgery. The patient was referred to the Rheumatology Department in the hospital for the aggravation of abdominal pain, involving the whole abdomen.

**Fig. 2:**
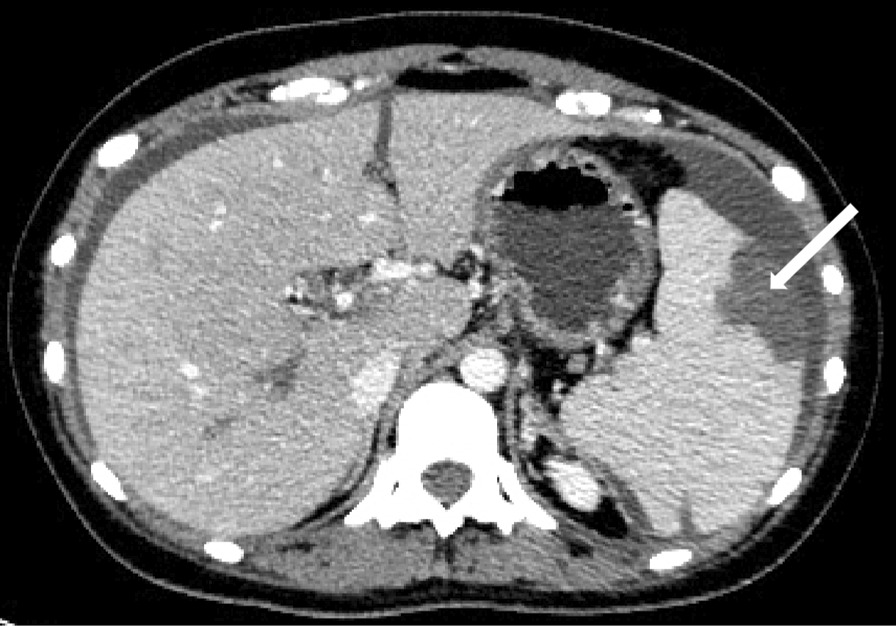
Wedge-shaped low-density lesion in spleen without enhancement (splenic in-farction)

The patient had two abnormal abortions in past medical history. Abnormal physical examinations included total abdominal tenderness, rebound pain and muscle tension. The patient declined for abdominal palpation. Laboratory investigations showed raised APL (anti-β2GPI 143R U/mL, ACL IgM 70 U/mL, ACL IgG 20 U/mL). Renal biochemistry, hepatobiliary biochemistry, serum lipid profile, muscular enzymes and thyroid function were normal. Computed tomography of the abdomen confirmed multiple thrombi in superior mesenteric vein and other vessels, dilatation of the small intestine and spleen infarction (Fig. 2).

The patient was diagnosed with antiphospholipid syndrome based on clinical manifestations, past medical history and auxiliary examination results. The patient did not meet the catastrophic antiphospholipid syndrome (CAPS) diagnostic criteria. The patient claimed a previous pregnancy loss and presented with characteristics of high-risk APL (Table 1). Moreover, the patient was affected by multiple abdominal vessels and viscus. Therefore, these findings demonstrated that the patient based on the 2019 EULAR recommendations for the management of antiphospholipid syndrome in adults, was at high risk. The patient was administered with intravenous immunoglobulin (gamma globulin 40 mg daily) and glucocorticoid (methylprednisolone 40 mg daily) to limit further disease progression. Low molecular weight heparin (LMWH, enoxaparin 40 mg twice a day) therapeutic dose was used for anticoagulant therapy. Abdominal pain was significantly improved after systemic treatment. The patient was discharged and put on warfarin given orally 3 mg daily, methylprednisolone 40 mg daily, and hydroxychloroquine 0.2 g twice daily to be taken with food.

**Table 1:** Antiphospholipid antibodies and other serological data of our patient

Antiphospholipid antibody	Titer
Anticardiolipin-IgG (U/mL) (< 12.0 U/mL)	20
Anticardiolipin-IgM (U/mL) (< 12.0 U/mL)	70
Anti-β2 glycoprotein (U/mL) (< 20.0 U/mL)	143
Lupus anticoagulant-dRVVT (ratio) (< 1.2)	1.49
Anti-SSA-52/Ro52	Positive
Anti-double-stranded DNA antibody (IIF)	Positive (1:320)

Characteristics of high-risk APL (1) ≥ 2 intervals of 12 weeks, presence of lupus anticoagulants (as determined by ISTH guidelines), (2) or the presence of any two or three aPL positive lupus anticoagulants, ACL, or anti-β2GPI, (3) or persistent high titer of APL

*IgG* immunoglobulin G, *IgM* immunoglobulin M, *dRVVT* dilute Russell viper venom time, *IIF* indirect immunofluorescence

**Fig. 3:**
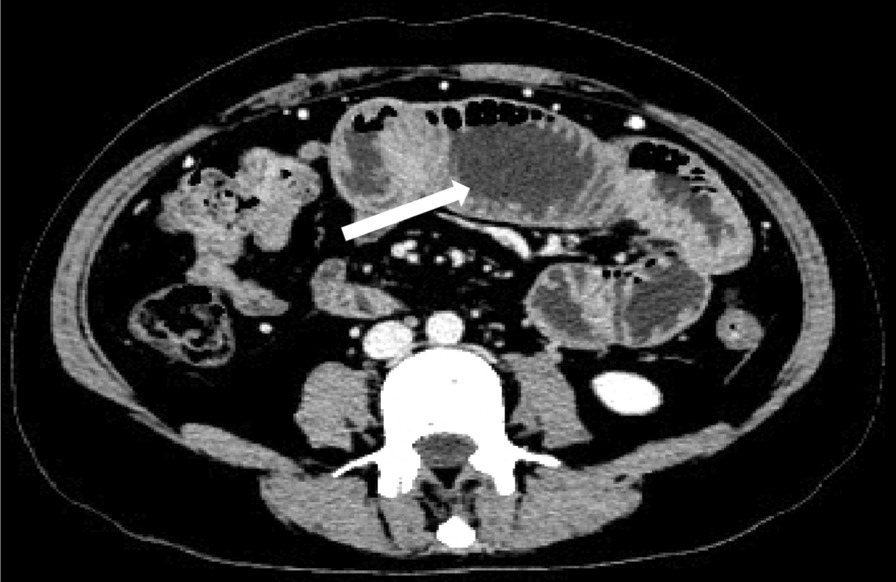
Incomplete intestinal obstruction, dilation and wall thinken of the small intestine

After months of discharge, the patient was later hospitalized with recurrent abdominal pain. The patient complained about nausea, vomiting, abdominal distension, no defecation, and no exhaust during the last treatment in the Rheumatology Department. The abdominal computed tomography indicated incomplete ileus (Fig. 3). Gastrointestinal decompression and conservative medical treatment were performed first. However, the therapeutic effect was not satisfactory. Therefore, the patient was transferred to the department of gastrointestinal surgery for surgical treatment. As for the reasons for the patient's admission, we consider the following two aspects: First, the patient had multiple mesenteric thromboses. Although anticoagulant therapy was applied, it could only prevent new thrombosis, the existing thrombosis could not be dissolved. Also, the collateral circulation was not completely established, leading to insufficient blood supply to the intestinal tract and non-mechanical obstruction. Second, although the patient regularly took warfarin anticoagulant therapy after discharge, the drug is greatly affected by diet and inconvenient monitoring, so the INR value outside the hospital was poorly controlled.

## Discussion and conclusion

APS is characterized by hypercoagulability of the blood. Thrombosis is the most common manifestation affecting blood vessels of any size. Therefore, APS is increasingly considered to be a multi-organ and multi-system disease with an expanding spectrum of clinical manifestations that may involve different organ systems [1].

The prevalence of APL in the general population ranges between 1 and 5% [7]. But only a minority of these people develop APS. According to the latest large population-based study, APS occurred in 2 persons per 100,000 population per year. The estimated prevalence was 50 per 100,000 population [8]. APS typically occurs in the fourth decade of life, and women are predominantly affected, especially in cases of secondary disease. Furthermore, there is no influence of race in APS [9].

The most common thrombosis events included stroke (5.3%), transient ischemic attack (4.7%), deep vein thrombosis (4.3%), and pulmonary embolism (3.5%) [6]. However, abdominal manifestations in APS patients are rarely reported. Among the APS patients, liver involvement is the most common manifestation which is followed by gastrointestinal tract [4].

Hamilton et al. reported a case in 1991 [10]. Related manifestations of gastrointestinal system have been reported successively, including esophagus [11], stomach [12], intestines [13], etc. Major related diseases include esophageal necrosis, esophageal rupture, gastric ulcer, intestinal infarction and inflammatory bowel disease.

The pathogenic mechanism is mainly manifested in two aspects, thrombosis and autoimmune. Organic thrombosis forms extensively in the gastrointestinal tract vessels due to the hypercoagulability of APS. This led to ischemic injury of the gastrointestinal mucosa and mucosal necrosis [11]. APS is an autoimmune disease that produces autoimmune antibodies in the body such as lupus anticoagulants. LA causes autoimmune esophagitis which reduced wall strength and increased the risk of spontaneous esophageal rupture [14].

Anticoagulation is the basis of treatment for APS patients. The choice of anticoagulation regimen should be based on the risk stratification of APS patients. Risk stratification includes characteristics of high-risk APL, history of thrombosis and/or obstetric APS, co-occurrence of other autoimmune diseases, and common risk factors for cardiovascular disease [3]. Prophylaxis with low-dose aspirin (75–100 mg daily) is recommended for asymptomatic APL patients which reduces the risk of adverse events such as antibody titers and thrombosis [3]. Additionally, high-risk asymptomatic APL carriers with traditional cardiovascular risk factors should be actively controlled to prevent accelerated atherosclerosis, APS artery thrombosis, lifestyle changes and drug therapy [3, 15]. Glucocorticoids, plasma exchange or intravenous immunoglobulins are not a routine treatment for APS. However, the combination of glucocorticoids, heparin, and plasma exchange or intravenous immunoglobulin is recommended as a first-line treatment for CAPS patients.

For APS patients with the above-mentioned abdominal manifestations, anticoagulation therapy has been applied for a long time. The International Normalized Ratio (INR) 2–3 or INR 3–4 is suggested for warfarin treatment [3]. Food and drug interference with increased risk of bleeding and compliance in APS patients with long-term use of warfarin as result of a regular laboratory testing is poor. Direct oral anticoagulants (DOAC, for instance Dabigatun, Rivaroxaban, Apisaban, idoxaban, etc.) inproves the situation.

DOAC is a single-enzyme anticoagulant drug which acts on the coagulation cascade. DOAC has the advantage of less interference with other drugs. It is not affected by diet and does not require routine INR evaluation [16]. Cochrane systematic review demonstrated that there is insufficient evidence to support or oppose the use of DOAC in thrombotic APS [17]. However, in recent randomized clinical trials of drugs and meta-analyses, APS patients taking DOAC had a higher risk of thromboembolism recurrence. These APS patients also had a higher incidence of major bleeding events than APS patients taking warfarin especially in patients with triple-positive APL [18–20]. Based on the above results, DOAC cannot be widely used in APS anticoagulant therapy. DOAC is only limited to patients with venous thrombosis whose INR cannot reach the standard after warfarin administration and patients with absolute contraindications of Warfarin [3].

Currently, there are new treatments for thrombotic APS, including anticoagulants supplemented with hydroxychloroquine, statins, vitamin D and sirolimus. Due to the effects of these drugs in thrombotic APS treatment there is a need for further studies [21].

Surgery should be performed immediately for patients with intestinal necrosis or perforation indicated by clinical symptoms, imaging or laboratory tests [22]. For doubtful patients, an open abdominal exploration should be performed to determine the viability of the bowel and thus the scope of resection [23]. Surgical reconstruction of the arteries appears to be feasible from published cases. However, further research is needed to establish optimal anticoagulation regimes and long-term management after surgery [24]. Moreover, large-scale clinical data with relevant retrospective analysis and other clinical studies are lacking to show whether surgical intervention brings long-term clinical benefits and improves long-term prognosis of patients with acute thrombus induced by APS.

Currently, there are no large or good controlled studies to guide clinical decision-making in terms of interventional therapy. Only a few successful cases of arterial thrombectomy have been reported [25–27]. There is no case of venous thrombectomy that has been reported. Additionally, eluting drug stent implantation is still controversial. A few studies cases have shown that intravenous/arterial thrombolytic therapy can successfully dissolve venous thrombosis [28] and effectively reduce acute macrovascular thrombosis [29].

This case report is meaningful because the patient presented with recurrent abdominal pain as the main clinical manifestation. Ischemic gastrointestinal manifestations implicated a rare initial presentation of antiphospholipid syndrome. The patient was finally diagnosed as APS after going to several hospitals and related departments. In this case, the patient's gastrointestinal vascular lesions were evaluated based on computed tomography. A recent study has shown that advanced magnetic resonance (MR) imaging can predict esophageal varices in patients with cirrhosis [30]. Based on the above research result, we believe that advanced MR imaging has great potential for further evaluation of gastrointestinal angiopathy in APS patients in the future. Anticoagulant regimen was strictly implemented for the treatment of this case. This was based on the above risk stratification of APS patients. The patient was administered with gamma globulin and methylprednisolone. However, the latest treatment advice does not mention intravenous immunoglobulin and glucocorticoid, considering the patient's past multiple abortions and the impact of multiple blood vessels and organs. The patient was transferred to gastrointestinal surgery for intestinal obstruction in cases where anticoagulant therapy failed to control the thrombotic progression and intestinal obstruction occurs.

In conclusion, APS-related gastrointestinal manifestations are diverse, complex, nonspecific, and easily confused with other abdominal diseases, which led to misdiagnosis. Therefore, other departments should have a proper understanding of APS. Cooperation among departments should be strengthened to achieve early detection, early diagnosis and early treatment to avoid delaying the initiation of treatment. Few cases of APS-associated gastrointestinal manifestations have been reported and the incidence was very low in many studies. However, the true incidence of APS-related gastrointestinal manifestations may be underestimated. A high suspicion index for any signs of abdominal involvement should be considered in patients with APS. Therefore, this report provides useful information on gastrointestinal manifestations and APS.

## Data Availability

All data generated or analyzed during this study are included in this published article.

## Abbreviations

APS: Antiphospholipid syndrome
APL: Antiphospholipid antibodies
LA: Lupus anticoagulants
Anti-β2GPI: Anti-β2-glycoprotein I antibodies
ACL: Anticardiolipin antibodies
CAPS: Catastrophic antiphospholipid syndrome
LMWH: Low molecular weight hepa-rin
INR: International Normalized Ratio
DOAC: Direct oral anticoagulants
